# A review and outlook on expression of animal proteins in plants

**DOI:** 10.3389/fpls.2024.1426239

**Published:** 2024-08-22

**Authors:** Daniel Tusé, Matthew McNulty, Karen A. McDonald, Leah W. Buchman

**Affiliations:** ^1^ DT/Consulting Group, Sacramento, CA, United States; ^2^ Center for Cellular Agriculture, Tufts University, Medford, MA, United States; ^3^ Department of Chemical Engineering and Global Healthshare Initiative, University of California, Davis, Davis, CA, United States; ^4^ Biotechniology Innovation Organization, Agriculture and Environment, Washington, DC, United States

**Keywords:** biotechnology, molecular farming, sustainability, regulatory, animal protein, safety

## Abstract

This review delves into the multifaceted technologies, benefits and considerations surrounding the expression of animal proteins in plants, emphasizing its potential role in advancing global nutrition, enhancing sustainability, while being mindful of the safety considerations. As the world’s population continues to grow and is projected to reach 9 billion people by 2050, there is a growing need for alternative protein sources that can meet nutritional demands while minimizing environmental impact. Plant expression of animal proteins is a cutting-edge biotechnology approach that allows crops to produce proteins traditionally derived from animals, offering a sustainable and resource-efficient manner of producing these proteins that diversifies protein production and increases food security. In the United States, it will be important for there to be clear guidance in order for these technologies to reach consumers. As consumer demand for sustainable and alternative food sources rise, biotechnologies can offer economic opportunities, making this emerging technology a key player in the market landscape.

## 1 Introduction and background

The global population increase presents a significant challenge in ensuring food security and meeting the diverse nutritional needs of humanity (FAO et al., 2019). This demographic expansion is accompanied by an evolution in dietary preferences across the world, influenced by urbanization, globalization, and cultural shifts. Alongside changes in dietary patterns, disparities in access to nutritious foods persist, exacerbating malnutrition and micronutrient deficiencies, particularly in low socioeconomic regions. The dynamics of population growth vary across regions; while some areas experience demographic expansion, others undergo demographic transitions characterized by declining birth rates and aging populations (Bongaarts, 2019). Shifts like these have implications for food systems, including all facets of the value chain from labor availability to market demand.

As the global population increases, meeting the food demand necessitates an expansion of agricultural production, raising concerns about environmental sustainability and resource depletion. Intensive agricultural practices, such as deforestation, monocropping, and excessive use of fertilizers and pesticides, contribute to biodiversity loss, soil degradation, and greenhouse gas emissions (Foley et al., 2011). Balancing the imperative for increased food production with environmental stewardship remains a challenge in achieving long-term food security. Achieving food security entails not only providing access to sufficient, safe and nutritious food but also addressing issues of affordability, stability, and sustainability throughout the food supply chain (FAO et al., 2023).

Despite decades-long advancements in agricultural productivity, disparities in food access persist across regions and socioeconomic groups. Vulnerable populations, including smallholder farmers, women, and marginalized communities, often face barriers to accessing nutritious foods due to factors such as poverty, limited infrastructure, and social inequalities (FAO et al., 2023). Food systems are impacted by climate change, natural disasters, conflicts, and economic fluctuations. These disruptions can impact food production, distribution, and access, exacerbating food insecurity and undermining livelihoods (Mbow et al., 2019). Building resilience within food systems requires investments in climate-smart agriculture, risk management strategies, and novel technologies to mitigate the stressors of climate change (FAO et al., 2023).

One approach to resolving these disparities is to broaden the sources of available dietary proteins, including adopting new platforms for producing traditional proteins using methods with improved resource utilization efficiency and environmental sustainability. In the past several years, increasing effort has been devoted to producing traditional animal proteins in plants via genetic modification of crops. Plant expression of animal proteins is a cutting-edge approach that allows crops to produce proteins traditionally derived from animals, enabling diversification of protein production and increasing food security.

This review explores the multifaceted benefits and considerations surrounding the production of animal proteins in plant systems, emphasizing the potential role of these systems in advancing global nutrition, and in enhancing sustainability while promoting environmental stewardship. Topics covered include the technologies supporting dietary protein production in crops, examples of development projects underway including commercial activity, comparisons of the nutritional quality and safety between animal- and plant-derived dietary proteins, the potential impact on public health of expressing allergenic proteins in crops as well as approaches to mitigating allergenicity, and the role of stakeholders – including growers, manufacturers, formulators, regulatory agencies, and consumers – in contributing to the adoption of such new protein sources.

As consumer demand for nutritious, safe, and sustainable alternative food sources continues to rise, these new approaches to dietary protein production can become a key component of the bioeconomy. For these efforts to succeed, fact-driven policies and clear and transparent communication strategies need to be implemented to keep consumers informed of the risks and benefits of the technologies employed and the new products they will yield.

### 1.1 Biotechnology and food security

Biotechnology comprises an important collection of tools for achieving global food security, offering innovative solutions to address key challenges in agricultural production, resource management, food quality and safety. Biotechnology encompasses a diverse array of techniques and methods aimed at harnessing biological processes to enhance crop productivity, improve resilience to environmental stresses, mitigate the impacts of pests and diseases, reduce the reliance on conventional chemical pesticides and minimize environmental impacts (Anderson et al., 2016).

For example, biotech-derived insect-resistant crops engineered to express insecticidal proteins from *Bacillus thuringiensis* (Bt) are effective in reducing crop damage and enhancing yields while minimizing chemical inputs (Tabashnik et al., 2013). Similarly, biotechnical tools enable the development of disease-resistant crop varieties through genetic modification or gene-editing techniques, providing sustainable solutions for managing plant pathogens and reducing yield losses (Dangl et al., 2013). Crops engineered for improved nutrient uptake, water-use efficiency and stress tolerance help optimize resource allocation and minimize waste, thereby enhancing productivity and conserving natural resources (Kamthan et al., 2016). Biotechnical tools also facilitate the development of bio-fertilizers, microbial inoculants, and biostimulants that promote plant growth, nutrient cycling, and soil health, fostering sustainable agricultural practices (Bhattaacharyya et al., 2015). Advances in biotechnology have led to varieties of crops with enhanced nutritional quality and safety, enabling us to address micronutrient deficiencies and improving dietary health outcomes. Biofortification strategies involving the enhancement of essential nutrients such as vitamins, minerals, and antioxidants in staple crops, contribute to combating malnutrition and improving public health, particularly in vulnerable populations (Bouis and Saltzman, 2017). Biotechnology is also an enabling tool for engineering plant hosts for *in vitro* (e.g., bioreactor-based) production of recombinant proteins and small molecules for a variety of applications ranging from food to pharmaceuticals. Leveraging biotechnical innovations in crop improvement, pest and disease management, resource use efficiency, bioreactor-based production and food quality enhancement can therefore contribute to building more sustainable, resilient, and equitable food systems to meet the challenges of a growing population and changing climate.

### 1.2 Role of technology in diversifying dietary protein sources

Technical advancements promoting dietary protein diversification can be broadly described as enabling: 1) novel processing options and/or utilization of an existing protein source, 2) introduction of new protein characteristics and functionalities to an existing protein source, and 3) production of a new or underutilized protein source. To date, most technical advances addressing dietary protein diversification at the commercial scale have focused on novel processing methods applied to existing protein sources. These methods have led to the development of plant-based protein alternatives that mimic the taste, texture, and nutritional profile of animal-derived proteins (Nowacka et al., 2023).

In addition, new approaches to dietary protein diversification include those aimed at modifying protein characteristics and as well as expanding the sources of dietary proteins. Microbial fermentation technologies allow the production of protein-rich ingredients from microorganisms such as fungi, algae, and bacteria. Traditional ideas, such as single-cell proteins derived from microbial fermentation (Tusé and Miller, 1983), are being revisited as they offer a scalable and resource-efficient source of protein with minimal environmental impact, and can enable partial replacement of animal-based protein demand (Pikaar et al., 2018). More recently, the concept of producing agricultural commodity goods (e.g., meat) through cell culture and tissue engineering (termed cellular agriculture), has emerged as a more efficient, sustainable, safe, and ethical alternative to conventional livestock farming (Post et al., 2020). In addition, a collection of techniques, generally referred to as Plant Molecular Farming (PMF) and discussed in the following sections, involves the use of genetically modified (GM) plants or plant cells/tissues- to produce a wide range of proteins for use in food, pharmaceutical, cosmetic, industrial and environmental applications (Ma et al., 2003; Fischer and Buyel, 2020). PMF methods also enable the precise modification of crops to produce protein-rich ingredients that can be utilized in alternative protein products, further diversifying protein sources and enhancing sustainability.

### 1.3 Reducing dependence on traditional livestock farming

Conventional livestock farming contributes to deforestation, greenhouse gas emissions, water pollution and biodiversity loss, exacerbating environmental degradation and climate change, and raises concerns about animal welfare including confinement, overcrowding, and routine use of antibiotics and hormones (Gerber et al., 2013). Introducing heritable genetic modifications in food animals presents opportunities for enhancing productivity, disease resistance, environmental sustainability (reduced methane emissions and increased heat tolerance) and improving animal welfare (Van Eenennaam, 2017). Although these are welcome steps, alternative technologies such as cellular agriculture and PMF also offer efficient and sustainable routes to producing animal proteins. By engineering crops to express dietary proteins, such as milk casein and whey and muscle proteins, these new techniques can provide a sustainable and ethical alternative to meet the increasing demand for nutritional proteins. These new approaches not only address environmental and welfare concerns associated with traditional animal agriculture, but also offer opportunities for creating animal protein alternatives with improved nutritional profiles, higher safety, and reduced environmental impact. For the foreseeable future, adoption of a combination of approaches that include improvements in animal agriculture as well as cellular agriculture and PMF are envisioned, as such a combination of complementary production formats will offer the best chance of ensuring sustainable food security.

### 1.4 Stakeholders in future value creation through alternative technologies

According to a report by the Good Food Institute (Battle et al., 2024), consumers continued to adopt plant-based meat, seafood, eggs, and dairy as healthy, sustainable alternatives to their conventional equivalents. Total global retail sales in 2023 reached $29 billion, up 34 percent from 2019. In addition, the market forecasts by 2035 for alternative proteins, including plant-derived, fermentation, and cultivated, range from $87 billion to $594 billion (Battle et al., 2024). Given the continued demand for animal proteins that are produced by more sustainable methods, the expression of animal proteins in plants could become a key component of this strategy.

Technology companies, including startups and established players, and research institutions worldwide are investing in research and development to create novel protein sources with improved taste and texture, and enhanced nutritional profiles and safety, with the goal of expanding the range of options available to consumers. These innovative groups see PMF as having the potential for revolutionizing the food industry and promoting sustainable protein production by leveraging the efficiencies and environmental benefits of plant-based production systems. Governments and regulatory agencies also play a crucial role in shaping the future of emerging biotechnologies, like alternative proteins through science-based, risk-proportionate policy development, funding initiatives, and regulatory frameworks (Schot and Steinmueller, 2018; Robinson et al., 2021; Buchman et al., 2024). Policies that incentivize sustainable food production, support research and development, and promote consumer education and awareness can facilitate the transition to a more diversified and sustainable food system. Ultimate, the success of these new protein production efforts will depend on consumer acceptance of new products, and such acceptance will be based on directly perceived organoleptic properties of novel foods and their nutritional value and safety, in addition to indirectly perceived attributes such as sustainability, access and equity. Therefore, collaboration among stakeholders across the food value chain, including producers, manufacturers, retailers, researchers, policymakers, as well as consumers, is essential for driving innovation, scaling up novel technologies and bringing new products to market.

## 2 Overview of plant expression of dietary proteins

In the various implementations of PMF (reviewed in Ma et al., 2003; Tschofen et al., 2016; Fischer and Buyel, 2020; Hefferon et al., 2023), specific genes of interest (GOI) are introduced to plants or plant cells and are then harnessed by the natural machinery of the plant host to produce complex and valuable products such as proteins. This can be achieved via stable integration of the GOI into the plant genome, in the case of transgenic production, or in a non-integrated impersistent fashion leading to eventual clearance of the GOI from the plant, in the case of transient production (Gleba et al., 2013; Schillberg and Finnern, 2021). For more than 30 years, both transgenic and transient methods of protein expression have been extensively studied to produce pharmaceutical proteins and vaccines as well as non-pharmaceutical proteins for use as food and feed ingredients and additives, cosmetics, and enzymes for food processing and biofuel production (reviewed in Gleba et al., 2013; Tusé et al., 2014; Tschofen et al., 2016; Buyel, 2019; Long et al., 2022 and others). Whole plants can be grown with varying degrees of containment ranging from open field cultivation to greenhouses to highly controlled indoor growth environments (e.g., controlled environment agriculture, CEA, or vertical farming). The materials and methods used in PMF have been surveyed and assessed to be generally safe and environmentally benign (Buyel, 2023).

PMF is considered a cost-effective, scalable, and environmentally friendly alternative to the predominant microbial or animal cell culture-based recombinant protein production platforms (Obembe et al., 2011). For whole plant molecular farming, the fundamental biomass production operations leverage a basic agricultural skillset as opposed to complex aseptic bioreactor training and skills, and the upstream processing of PMF is also perceived as a better skills match and route to increase accessibility of pharmaceutical and healthcare options for lower- and middle-income countries (Murad et al., 2020). The same is expected to be true in the production of dietary proteins.

Recently, PMF has been receiving increasing industry attention as a platform for the production of dietary proteins (Long et al., 2022). The set of constraints around food protein production are well aligned with PMF’s strengths, including 1) the relatively lower margin and higher volume production requirements are well met by PMF’s lower cost and ease of scalability; 2) the end-product requirements for human consumption are met by a range of PMF host organisms that are Generally Recognized as Safe (GRAS) by the U.S. Food and Drug Administration (FDA); and 3) the relatively lower purity requirements for human food ingredients and additives relative to pharmaceutical products (e.g., for intravenous injection) reduces the downstream processing requirements, the costs of which for PMF have traditionally been viewed as a barrier to wider market penetration (Schillberg and Finnern, 2021). Additionally, a considerable portion of the food proteins recently considered for PMF production are animal proteins, the production requirements of which align with the strengths of the PMF platform and benefit from what has been learned in the production of animal-derived pharmaceutical and industrial proteins.

### 2.1 Whole-plant systems and current development programs for producing dietary animal proteins in plants

Parallel approaches in PMF can be applied upstream to produce recombinant proteins, and generally consist of whole plant systems grown in open fields or indoors (discussed here), or of plant cells and tissues cultured in contained environments (Section 2.2). The whole-plant approach can take full advantage of agricultural economies of scale. Biomass production upstream, especially in open fields, does not require the often-costly capital investments required for contained systems thereby enabling significant upstream processing (USP) cost savings. In addition, technical improvements made during the last decade (e.g., centrifugation, filtration, flocculation, two-phase separation systems, protein purification tags, membrane technologies and heat/pH precipitation) have led to reductions in downstream processing (DSP) costs, which have traditionally been a major cost driver in PMF.

Using both transgenic and transient PMF approaches (reviewed in Tschofen et al., 2016; Hefferon et al., 2023), plants could be viable sources of animal-free dairy, egg, meat and seafood proteins, as well as human milk proteins, with high potential for scalability and a more environmentally benign footprint than animal-based food production methods (Panescu et al., 2023).

As mentioned, PMF has already been applied to produce a wide variety of protein-containing products, including pharmaceutical proteins and vaccines and proteins for use as food and feed ingredients and additives and food processing reagents, among other uses. Based on this body of work in PMF, the diverse efforts to express animal proteins in plants by various companies are exemplified in **Table 1**.

**Table 1:** Partial List of Companies Producing Animal Proteins in Plants.

Company (headquarters) Alphabetical listing	Protein Source (Expression Host Plant)	Comments
Forte Protein (USA)	Lactoferrin, casein, albumen, collagen, myosin and other proteins (lettuce, kale, spinach and other crops)	Meat, fish and dairy proteins expressed transiently in plants for multiple animal-free food applications
IngredientWerks (USA)	Bovine myoglobin (corn)	Bovine muscle protein as isolated ingredient or *in-matrix* use for plant-based meat
InVitria (USA)	Human lactoferrin, human lysozyme, human albumin, human transferrin (rice)	Human proteins produced recombinantly in plants for the research and cultivated products markets
Miruku (New Zealand)	Dairy proteins and fats (oilseed crops – unspecified)	Animal-free dairy ingredients
Mozza Foods (USA)	Casein (soy)	Recombinant soy expressing bovine casein proteins for conversion into lactose-free, non-animal cheese
Moolec Science (Luxembourg)	Porcine and bovine proteins such as myoglobin (pea, soy) Ovum (wheat) Whey (oat) Chymosin (aka rennin; in safflower)	Taste and functionality added to pea and soy seeds. Company reported expression of porcine myoglobin in soybeans at more than 26% of total soluble protein. Plant-made chymosin (rennin) developed with Bioceres (Argentina) for animal rennet-free cheesemaking
NewMoo (Israel)	Multiple bovine casein proteins expressed in soybean and other crops	Instead of purified casein powders, initial product consists of liquid casein obtained with minimal processing and having functionalities similar to milk, for the animal-free cheese market
Nobell Foods (USA)	Casein (soy)	Recombinant soy expressing bovine casein proteins for conversion into lactose-free, animal-free cheese
ORF Genetics (Iceland)	Growth factors (barley seed)	Recombinant barley seeds expressing endotoxin-free bovine, porcine, and avian growth factors for cultivated meat applications
Pigmentum Ltd (Israel)	Casein (lettuce)	Multiple casein subtypes, including αs1-, αs2, β- and κ-casein expressed in transgenic lettuce for alternative dairy sector
PoLoPo (Israel)	Ovalbumin (potato)	Functionally equivalent to egg albumin protein; product is intended for non-animal food ingredients market
Spidey Tek (USA)	Spider silk (alfalfa)	Plant-grown spider silk proteins for use in adhesives, cosmetics, structural composites, and other applications requiring strong yet light-weight components
Tiamat Sciences (Belgium & USA)	Growth factors and other animal proteins (unspecified)	Animal- and endotoxin-free growth factors produced transiently in plants for use in cultivated meat production

Many of these companies began with a focus on plant-expressed dairy and egg components, while others have broader pipelines that include expressing meat-associated proteins such as myosin and myoglobin. Some are producing in plants media components for use in cultivated meat production, and at least one company is producing animal-derived structural proteins (e.g., spider silk).

Although production of dietary and other proteins in whole plants is favored due to better production economies, whole-plant PMF, especially when practiced in open fields, offers economic advantages at the expense of control and containment. Containment is important when growing GM crops in the vicinity of the same species of non-GM crops, as cross pollination could take place thereby potentially adulterating both the GM and non-GM products. Also, care must be taken to prevent harvesting and stewardship errors that could result in exposure of consumers to unexpected proteins, with potential health consequences. This topic is further addressed in Section 5.7. When higher process control is desired, contained systems for whole plants ranging in complexity from greenhouses to indoor vertical farming, and plant tissue and cell culture approaches, can offer viable alternatives for producing specific proteins.

### 2.2 Plant cell bioreactors for producing recombinant proteins and edible biomass for food applications

Transgenic plant cells, embryos, hairy roots, moss, and aquatic plants can be grown *in vitro* in closed bioreactor systems to produce a variety of recombinant proteins, including animal proteins, and edible plant biomass for food applications. These types of systems offer some advantages over transgenic whole-plant production as well as bioreactor-based production of recombinant animal proteins using microbial or animal cell culture. Because bioreactors are inherently closed, fully contained systems that generally require aseptic operation, free of environmental contaminants (e.g., heavy metals, microbial pathogens, etc.) and can be operated in compliance with food good manufacturing practice (GMP) guidelines, regulatory concerns related to food safety, cross-contamination of other food or feed streams, and environmental release of GMOs (Dietz and Muldoon-Jacobs, 2024) are alleviated. Of course, these production systems need to meet food quality and safety requirements, including absence of food allergens and toxic compounds.

One specific concern regarding plant cell culture is the common use of synthetic plant growth regulators, such as 2,4-D (an agricultural herbicide regulated by law in the U.S. and Europe), in laboratory plant cell culture media. These components will need to be substituted with food-safe alternatives for food applications. Additional considerations for food safety of plant cell culture food products can be found in Häkkinen et al., 2020.

But unlike many crop-based production systems, plant cell bioreactors can operate year-round, anywhere in the world (or even in space), potentially with a smaller footprint, and the ability to precisely control culture conditions (T, media composition, dissolved oxygen, hydrodynamic shear environment, etc.) allowing better batch-to-batch reproducibility, the potential to optimize productivity, and to influence product quality. In addition to optimizing bioreactor environmental conditions, a wide variety of operating strategies including batch, fed-batch, semicontinuous (fill and draw), perfusion and continuous cultures can be implemented, depending on whether the recombinant product is intracellular or secreted (and stable) in the culture medium.

Compared with microbial or animal cell culture, transgenic plant bioreactor systems offer other benefits, including the ability to grow cells on low cost, chemically-defined, animal component-free media, relative robustness to perturbations in environmental conditions, and lower oxygen uptake rates that reduce equipment requirements for oxygen mass transfer. In addition, since animal viruses and phages don’t propagate in plants, there is lower risk of manufacturing disruptions and/or product safety risks from contamination by these pathogens. In some cases, for example in the moss (Decker and Reski, 2020) and *Lemna* (Coughlan et al., 2022) production systems, the plant hosts can be grown photosynthetically under natural or artificial light, thereby reducing the demand for sugars. Although the growth rate of plant hosts in these systems is lower than for microbial and/or animal cell culture, once a sufficient cell density is achieved, these systems are more amenable to long term production in semicontinuous, continuous or perfusion operations. For example, transgenic rice cell bioreactors have been operated continuously for over 80 days with multiple recombinant protein production cycles (Macharoen et al., 2021). Sustained production over long time periods has many advantages. Not only does it lead to higher productivity (reducing “turnaround times” for harvesting, cleaning, sterilization, inoculation and growth), but it also lowers cost (reduces energy for sterilization cycles, cleaning solutions, and number of seed train operations) and environmental impact (water use, disposal of cleaning solutions, generation of steam/energy). And importantly, it also allows for the use of single use disposable bioreactors (Maschke et al., 2024) in a more cost-effective and sustainable way since they can be used over very long time periods (e.g., 6-12 months). This feature enables distributed, “scaled out” production systems (larger numbers of distributed smaller sized bioreactors) compared with scaled up systems (localized facilities with larger working volume bioreactors).

Lastly, in all of these systems because the plant biomass is inherently multicellular and relatively large, ranging from hundreds of microns for plant cell cultures (which grow as small aggregates of cells rather than single cells) to millimeters for embryos and aquatic plants to centimeters for hairy roots and moss, they can be easily separated from the medium using gravity sedimentation in the bioreactor or low speed centrifugation. This can be an advantage for recovery of products secreted to the culture medium, compared with cultures that utilize single cells (e.g., bacteria, yeast, animal cells, as well as some algal and fungal cultures), where high speed centrifugation and/or microfiltration unit operations are required to recover the product.

There are some disadvantages to using plant bioreactor production systems compared with whole-plant PMF approaches. Plant cell/tissue/organ bioreactors have higher upstream production costs and environmental impact due to containment (bioreactors) and aseptic operation (cleaning/sterilization), a more complex supply chain, and scale-up challenges that all bioreactor-based systems have as mixing, mass transfer and heat transfer properties change during scaleup. In addition, there are still plant host cell line development challenges in terms low volumetric productivity, production stability (due to gene silencing, epigenetic modifications, somaclonal variation, and host proteolytic enzymes), long times required to screen many independent transformation events to identify highly productive cell lines, efficient product secretion, and cryopreservation methods for cell banking. Feng et al. (2022) describe molecular biology strategies that can be used to increase recombinant protein production in plants. It should also be noted that, in many cases, transgenic plant cell cultures can be established directly from explants from transgenic plants using standard plant tissue culture methods, typically within a few months. Plant-based cell-free protein production systems, such as the commercial ALiCE^®^ system that utilizes tobacco BY-2 cell extracts (LenioBio, GmbH), are being developed and scaled up into bioreactors (Gupta et al., 2023), but these systems will still rely on bioreactor production for production of the plant cells.

There are many publications describing production of animal proteins *in vitro* using plant cell hosts, and at least one commercial product, Elelyso™ made in carrot cell culture (Fox, 2012; Tekoah et al., 2015), for human health applications (therapeutics, nutraceuticals, and dietary supplements). Plant cell culture is also used for production of cosmetics, flavorings, and colorants (see reviews by Schillberg et al., 2013; Davies and Deroles, 2014; Gubser et al., 2021; Krasteva et al., 2021; Barzee et al., 2022), but there are fewer examples for bulk food applications, likely due to the higher production costs, as well as other technical challenges described above. There are also numerous examples of heterologous plant proteins produced in transgenic plant cell bioreactor systems for food applications such as the sweetener miraculin in transgenic carrot cell cultures (Park et al., 2020).

**Table 2** provides a few examples from published work that utilize plant cell bioreactors for production of animal proteins or plant biomass for food applications. In the latter category, there are additional opportunities for expression of animal proteins such as those described in Section 2.1 in these systems for enhanced nutrition, texture, or flavors.

**Table 2:** Examples of Animal Proteins and Plant Biomass Produced *In Vitro* for Food Applications.

Target/Application	Plant host/type	References
Basic Fibroblast Growth Factor (bFGF)/Cultivated meat media	Rice (*Oryzae sativa*)/cell suspension culture	Karmaker et al., 2024 Jia et al., 2024
Plant cell culture berries/Food	Cloudberry (*Rubus chamaemorus*), lingonberry (*Vaccinium vitis-idaea*) and stoneberry (*Rubus saxatilis*)/cell suspension cultures	Nordlund et al., 2018
Plant cell culture coffee/Beverage	Coffee *(Coffea arabica)*/cell suspension culture	Aisala et al., 2023
Plant cell culture chocolate/Food	Cocoa (*Theobroma cacao*)/cell suspension cultures	Eibl et al., 2018
Edible aquatic plants/Food	Duckweed species: *Spirodela, Landoltia, Lemna, Wolffiella and Wolffia/*shake flask cultures under artificial light	Appenroth et al., 2017

## 3 Product and facility classifications

It is important to consider the different ways in which PMF of animal proteins can be practiced as a technology to meet food industry needs and how production economics (and regulatory stewardship) will vary according to the selected product and process characteristics. Important dimensions to consider in evaluating PMF-based production economics for food applications include: 1) production volume per year, 2) product formulation (*in planta*, purified to some degree from the host plant substrate), 3) product application (end-product for direct human exposure/consumption, raw material for or input to food biomanufacturing), 4) process characteristics (e.g., plant host, expression modality, whole-plant vs. plant cell culture), and 5) facility classification (controlled indoor cultivation, semi-controlled indoor greenhouse cultivation, open-field cultivation, contained cell culture-based biomanufacturing facility).

To illustrate the impact potential of these dimensions, here we further detail considerations of product formulation, which will impact the production costs most distinctly through downstream processing contributions. A report on techno-economic analysis of therapeutic monoclonal antibody production in *N. benthamiana* using transient agroinfiltration and hydroponically grown plants found that 65% of total costs were associated with downstream operations to purify the target animal protein from the plant host (Nandi et al., 2016). Most of these costs would not have been necessary for *in planta* formulation, which does not require product purification from the host plant. A pharmaceutical purification process can be loosely considered an upper bound on the downstream processing contributions, given the more stringent purity requirements (>99% in this report) of the pharmaceutical GMP relative to food GMP purity standards. In a techno-economic report on production of food-grade antimicrobial proteins with lower purity requirements (>92%), the base case downstream processing contributed 42% of operating costs (McNulty et al., 2019). In this way, differences in product application, and thus differences in the quality standards for the process and product, as governed by pharmaceutical GMP and food GMP in the two example reports discussed, also influence the product formulation requirements and production economics.

The techno-economic study on antimicrobial proteins also illustrates the impact that facility classification can have on the production economics; the outdoor field-cultivation model reduced cost of goods sold estimates by 56% compared to the controlled indoor cultivation base case model. It is important to note that the outdoor field-cultivation model did not account for upstream difficulties of outdoor climate exposure including crop loss due to adverse weather events or the variability in both plant growth and product accumulation.

## 4 Equitable distribution and economic opportunities

The production economics of animal protein expression in plants will be a key consideration in when and how this production platform is widely adopted for food applications. PMF is a promising production platform for meeting the current and future food system needs for sustainable proteins, growth factors, and value-added ingredients. A majority of the emerging interest for PMF in food is to support the growing industry of alternative proteins, the umbrella term for food proteins sustainably and efficiently produced using plants, animal cell culture, and fermentation that are designed to taste as good or better than animal livestock products. Alternative protein products include plant-based meat analogs (e.g., Impossible™ Beef), cultivated meat (UPSIDE Foods’ cultivated chicken fillet), and animal-free dairy (e.g., Perfect Day’s ProFerm™ whey protein). Alternative proteins are creating unprecedently high-volume and low-cost demands for traditionally lower-volume animal proteins and openings for entirely novel animal protein needs. For example, a recent techno-economic analysis of cultivated beef production facility using an assumption basis of 100,000,000 kg of beef biomass per year (~0.14% annual global beef production; equivalent to an average U.S. slaughterhouse) (Negulescu et al., 2023) reported an annual demand of 20,500 kg recombinant transferrin. This single facility represents a demand that exceeds current global production capacity of transferrin by 70-100x (200 – 300 kg) (Market Research Report No. 197746, 2021).

## 5 Nutritional and safety considerations regarding expression of new dietary proteins in plants

### 5.1 Animal proteins, plant proteins, and combinations thereof

With respect to nutritional quality, animal-sourced dietary proteins have a higher content of essential amino acids than plant proteins, explaining in part the increasing demand for animal meat: terrestrial animal-sourced foods provide more energy and essential nutrients than other food types (FAO, 2023). There are other notable differences between plant and animal dietary proteins. In addition to their amino acid composition, they differ in structure, digestibility, and physicochemical functionalities. These factors can impact protein bioavailability, sensory and nutritional qualities (Day et al., 2022).

Development efforts to date have focused on producing known, native animal-sourced proteins in plants, with the goals of either extracting and purifying the protein after harvest for use as a food ingredient or supplement, or of co-expressing and accumulating the animal protein along with native plant proteins for consumption in edible plant biomass (leaves or seeds) with enhanced nutritional content. A benefit of the latter biofortification approach is to also impart on edible plant biomass new textures and flavors to improve the functional qualities of plant-based foods.

### 5.2 Animal vs. plant proteins: nutritional considerations

Compared to animal proteins, plant-based foods typically provide incomplete protein nutrition due to lower digestibility and plant-specific deficiencies in essential amino acids. Such differences may be more consequential to the dietary and developmental needs of infants and young children, due to the more restricted range of foods they can consume relative to adults (Balandrán-Quintana et al., 2019; Day et al., 2022). The lower digestibility of plant proteins is due in part to their higher hydrophobicity, propensity for aggregation and lower flexibility relative to animal proteins (Balandrán-Quintana et al., 2019; Zhao et al., 2020; Zhou et al., 2021).

The nutritional quality of a protein is determined by its essential amino acid content, protein digestibility, net protein utilization, biological value, and protein digestibility-corrected amino acid score (PDCAAS; FAO/WHO, 1991). The PDCAAS is an indicator of overall protein nutritional quality and can be used to compare proteins from various sources, with a score of 100 considered ideal. Animal-sourced proteins consistently score higher than plant-sourced proteins. For example, the PDCAAS is 92 for beef, 91 for chicken, and 100 for egg, whole milk and whey proteins. In contrast, the scores for plant-derived proteins are 93 for soy flour, 64 for yellow split pea, 74 for chickpea, 51 for wheat and 25 for wheat gluten (Ismail et al., 2020).

These deficiencies can be overcome by consuming proteins from multiple sources with complimentary amino acid profiles. But while a diet consisting purely or largely of vegetable proteins may be less desirable nutritionally, consuming a higher proportion of plant proteins in a balanced diet has been linked to several health benefits, including reduction in cardiovascular disease and certain cancers associated with fat and cholesterol in diets that are high in animal meat (Naghshi et al., 2020).

Collectively, these studies suggest that the co-expression of animal proteins in crops in concert with native plant proteins could solve the nutritional imbalance and lower digestibility of typical plant proteins, provide the benefits inherent in animal proteins without the less healthy components of animal meat, especially red meat (e.g., saturated fat, cholesterol), while bypassing the inefficiencies and ethical issues of animal agriculture. As summarized in **Table 1**, these efforts are already underway.

### 5.3 Safety considerations when expressing new dietary proteins in plants

Although nutritional improvements imparted on foods along with new or enhanced functional and organoleptic properties are clear potential benefits offered by the programs discussed here, one safety concern regarding expression of new dietary proteins in plants is the potential for cross contamination of the food supply with an allergenic protein. Recombinant crops expressing animal dietary proteins must be tracked throughout all steps of production, including plant cultivation, product extraction, transport, storage, and distribution, to prevent commingling with non-GM crops. Other safety concerns in PMF include potentially deleterious environmental impacts of field-propagated GM crops, risk from bacterial and viral vectors used in transient expression campaigns, plant host-derived impurities and/or secondary plant metabolites that may remain in the final product, process-related toxic impurities such as endotoxins, and other host-, product- and process-specific factors. It is important to emphasize that those risks and others were examined in a recent review of the safety aspects of PMF (Buyel, 2023), which concluded that none of the risks were sufficiently significant to prevent the use of plants in PMF applications in the food chain.

The safety of the proteins themselves expressed in heterologous systems is primarily tied to the allergenic potential of the introduced proteins, the potential for plant-specific post-translational modifications that could generate altered proteins, as well as their immunological cross-reactivity with other food allergens. Dietary proteins from both plant and animal origin can be allergenic; however, only a few protein families currently account for the majority (>90%) of food allergies (Costa et al., 2022a, b).

### 5.4 Dietary proteins and allergenicity

Food allergies are a worldwide problem, are increasing in incidence, and are as significant as malnutrition with respect to health impacts, with an estimated 10% of the world’s general population (~800 million people) afflicted by one or more food allergies (Broekman et al., 2015; Tanno and Demoly, 2022). Notably, approximately 20–65% of patients with irritable bowel syndrome (IBS) also report food-allergy related symptoms (Singh and Makharia, 2020).

Food allergy is an adverse health effect caused by a failure to establish or maintain oral tolerance to food proteins, is mediated by several types of immune system cells, and is influenced by the intestinal microbiota. Food allergy arises from specific immune responses to an allergen, whereas nonallergic adverse reactions to foods may be the result of food intolerances or metabolic deficiencies (Nowak-Węgrzyn et al., 2022). Typically, ingestion of dietary proteins in foods and other environmental “non-self” molecules leads to immune tolerization so that exposure does not lead to harm. The regulation of oral tolerance versus allergy is mediated in part by antigen-presenting cells, including dendritic cells, monocytes and macrophages, which present food antigens to CD4+ T cells and depending on tissue-specific signals either drive expansion of T-helper type 2 cells and promote IgE-specific B-cell responses (allergy), or expansion of regulatory T cells (tolerance) (Olivera et al., 2021).

The major allergenic proteins in the most widely consumed animal and plant foods are summarized in **Table 3**. The list is derived from the United Nations’ Food and Agriculture Organization (FAO) 1995 priority list of 8 food allergens, which included milk, egg, fish, crustaceans, tree nuts, peanut, wheat, and soybean, that was subsequently included in the General Standard for the Labeling of Packaged Foods (GSLPF) in 1999 (Wiederstein et al., 2023). The FDA adopted the same 8 foods in the GSLPF list through the Food Allergen Labeling and Consumer Protection Act of 2004 (FALCPA), and recently added sesame to the list through adoption of the U.S. Food Allergy Safety, Treatment, Education, and Research (FASTER) Act, which became effective on January 1, 2023.

**Table 3:** Allergenic Proteins in Widely Consumed Animal- and Plant-Based Foods.

Food Source	Allergen(s)	Prevalence; Comments	Representative References
Cow milk	Of the more than 20 proteins in cow’s milk, the primary allergens include caseins (alpha-s1-, alpha-s2-, beta-, and kappa-casein) and whey proteins (alpha-and beta-lactoglobulin)	In the developed world, the prevalence appears to be 2 to 3% in infants, falling to under 1% after 6 years of age. Most individuals with cow’s milk allergies are sensitive to both caseins and whey proteins. Reactions are IgE- and non-IgE mediated	Lifschitz and Szajewska, 2015 Edwards and Younus, 2023
Eggs	Most egg allergens are in the egg white fraction, including ovomucoid (Gal d 1), ovalbumin (Gal d 2), ovotransferrin (Gal d 3), lysozyme (Gal d 4), and ovomucin, although lipocalin-type prostaglandin D synthase and egg white cystatin are also associated with IgE reactivity. Hen egg yolk also contains allergens, the major one being alpha-livetin (Gal d 5)	US prevalence is difficult to establish accurately due to nonuniform diagnostic criteria. In studies in Scandinavia, egg allergy ranged from 1.6% to 2.2%, with 54% being IgE-mediated and 46% non-IgE-mediated	Boyce et al., 2010 Mathew and Pfleghaar, 2023
Fish	Most commonly consumed fish species contain allergenic parvalbumin proteins, which are known to cause fish allergies. More than 95% of all fish-induced food allergies are caused by parvalbumins	Prevalence of fish allergy is 0.2% for children and 0.5% for adults and is higher in women than in men, at 0.6% vs 0.2%, respectively. Reactions are typically IgE-mediated	Boyce et al., 2010 Mukherjee et al., 2023
Crustaceans	Multiple allergenic proteins in crustacean shellfish can lead to allergic reactions, including tropomyosin (the major allergen across all edible crustacean species), arginine kinase, myosin light chain, sarcoplasmic calcium binding protein, troponin C and triosephosphate isomerase	Allergy to crustacean shellfish in the USA is 0.5% for children and 2.5% for adults, and prevalence is higher in women than in men, at 2.6% vs 1.5%, respectively. Reactions are typically IgE-mediated	Boyce et al., 2010 Lopata et al., 2016
Tree nuts	Most proteins involved in tree nut allergy belong to protein families of 2S albumins, 7S globulins (legumins), 11S globulins (vicilins), non-specific lipid transfer protein (nsLTP), pathogenesis-related (PR)-10, profilins and oleosins	Prevalence of tree nut allergy in the USA is 0.4% to 0.5% of the population. Prevalence of tree nut allergy in France, Germany, Israel, Sweden, and the United Kingdom varies between 0.03% and 8.5%. Reactions are typically IgE-mediated. Tree nut allergy accounts for 18-40% of all cases of food-borne anaphylaxis	Boyce et al., 2010 Weinberger and Sicherer, 2018 Borres et al., 2022
Peanut	Peanuts harbor 12 allergens with multiple isoforms, which can be categorized into four of the most common food allergen families: Cupin superfamily (Ara h 1, 3), Prolamin superfamily (Ara h 2, 6, 7, 9), the Profilin family (Ara h 5), and Bet v-1-related proteins (Ara h 8), plus two additional families, Oleosin (Ara h 10,11) and Defensin (Ara h 12, 13)	Peanut allergy prevalence in the USA has been estimated to range from 0.6% to as high as 2% of the population. Prevalence in France, Germany, Israel, Sweden, and the United Kingdom varies between 0.06% and 5.9%. Peanut allergy is an IgE-mediated type 1 hypersensitivity reaction. Tree nuts and peanuts account for 70-90% of food-related anaphylactic fatalities	Boyce et al., 2010 Mueller et al., 2014 Weinberger and Sicherer, 2018
Wheat	Four groups of proteins are linked to allergic reactions to wheat. These include gluten proteins, including alcohol-soluble gliadins (or prolamins; 30%-40%) and acetic acid or alkali-soluble glutenins (or glutelins; 45%-50%), and non-gluten proteins, such as water-soluble albumins (10%–12%) and saline-soluble globulins (5%–8%).	Worldwide prevalence of allergies to wheat proteins is estimated as 0.5% to 9%. In the USA, prevalence is 0.4% for adults and 0.4-1% for children. Estimates for the European Union range from 0.3% to 3.4%. Allergic reactions to wheat proteins are both IgE- and non-IgE-mediated. Celiac disease (1-2% prevalence in the USA) is an example of a non-IgE-mediated pathology	Leonard and Vasagar, 2014 Jin et al., 2019 Patel and Samant, 2023
Soybean	Soybeans contain at least 16 different proteins, of which the storage proteins β-conglycinin (7S, Gly m 5) and glycinin (11S, Gly m 6) are the two most abundant, accounting for 70%–80% of the total seed globulin fraction. These two proteins, plus Gly m Bd 30 K (P34), Gly m Bd 28 K, and Gly m 4 17 K constitute the main dietary allergens in soybeans	Worldwide prevalence of allergies to soybean ranges from 0.3% to 3%. Most allergic responses to soy proteins are non-IgE-mediated, occur in younger children, and can include eosinophilic esophagitis	Mulalapele and Xi, 2021; Wang et al., 2022 Wiederstein et al., 2023
Sesame	Although sesame seeds contain some lipid allergens, multiple proteins are associated with allergic reactions, including albumins, globulins, oleosins, and one vicilin-like globulin. Of these, the main allergenic proteins include hydrophilic allergens (Ses i 1, 2, 3, 6, 7 and 8) and hydrophobic allergens (oleosins Ses i 4 and 5).	In a recent survey, 0.49% of the U.S. population reported a current sesame allergy, whereas 0.23% met symptom-report criteria for IgE-mediated allergy. Among individuals with verified IgE-mediated sesame allergy, an estimated 23.6% to 37.2% had previously experienced a severe allergic reaction, depending on the definition used. Other surveys estimated the prevalence in the range of 0.1–0.2% in the USA and Western Europe, and as high as 0.8–0.9% in the Middle East and other regions where sesame seeds are widely consumed	Adatia et al., 2017 Gangur and Acharya, 2021

Unless a protein is altered when it is expressed in a plant, the transfer of non- or hypo-allergenic animal proteins to plants is not expected to lead to significant risk. However, immune reactions in sensitive individuals could be expected if they consume plants expressing known allergenic animal proteins, or allergenic plant proteins expressed in a new host. Allergic reactions to these proteins can be generally classified into groups of pathologies, both IgE-mediated and non-IgE-mediated. Depending on an individual’s sensitivities, such reactions may have a rapid onset and can lead to multi-organ system anaphylaxis, which can be life-threatening, or to lesser symptoms that may be localized. Such adverse reactions have been characterized in original studies and reviews (e.g., Boyce et al., 2010; Mobayed and Ali Al-Nesf, 2014; Zhang et al., 2021).

### 5.5 Plant glycans and their role in allergenicity

It has been established that the carbohydrate component in plant glycoproteins can be responsible for the development of food allergies, in particular through the presence of β(1,2) xylose and α(1,3) fucose moieties (Garcia-Casado et al., 1996; Fu et al., 2023). Although both animals and plants have pathways for N- and O-glycosylation of proteins, the resultant glycoforms are different, absent, or present in plants but not animals. One particular type of allergy to mammalian meat is caused by pre-exposure to the glycan galactose-alpha-1,3-galactose (alpha-Gal for short), typically through a tick bite. Antibodies against the sugar can then lead to delayed hypersensitivity reactions to alpha-Gal on meat that can range from mild to life-threatening. Fortunately, alpha-Gal is not found in plants (Butler and Spearman, 2014; Dicker and Strasser, 2015; Román-Carrasco et al., 2020).

However, some types of plant glycan structures can lead to allergic and other adverse immune reactions to dietary proteins, including reactions to animal and human glycoproteins expressed in plants. In fact, the development by several groups of plant hosts devoid of, or containing inactivated genes for, β(1,2)-xylosyltransferase and α(1,3)-fucosyltransferase, singly or in combination with other manipulations of plant metabolic pathways, has enabled the production of human and animal therapeutic glycoproteins and vaccines with much lower allergenic or immunoreactive potential (Mercx et al., 2017; Montero-Morales and Steinkellner, 2018; Eidenberger et al., 2023). *In vitro* modification of glycans on purified proteins (Zhang et al, 2023) offers a flexible alternative, but may incur additional costs.

Human immune responses to plant glycans are contextual and may depend on the route of exposure. For example, in two separate studies of plant-produced recombinant vaccines delivered subcutaneously without or with an adjuvant, the polypeptide antigens containing plant-type glycans generated the expected antigen-specific immune responses, but no responses were directed to the glycan moieties on the glycoproteins (McCormick et al., 2008; Tusé et al., 2015). These results differ from the known immune responses to dietary plant glycoproteins (see above references) and may reflect differences in antigen processing and immune response mechanisms between the parenteral and oral routes of exposure. Nevertheless, the concerns relevant to this discussion are the potential consequences of expressing animal-derived dietary glycoproteins (e.g., casein, ovalbumin) in a plant host and generating altered glycoproteins of unknown allergenicity.

### 5.6 Allergen cross-reactivity among dietary proteins

In addition to species- and protein-specific allergens, allergy to one food can lead to cross-reactions to other types of foods. Such cross-reactivity can be especially important either when expressing animal proteins in plants for extraction, or when co-expressing animal proteins with native plant proteins in edible crops for consumption. Many proteins in candidate expression host plants are conserved across species and share allergenic epitopes. For example, the 2S albumin proteins in sesame seeds partially share amino acid sequence and structure with 2S albumin proteins from other plants. These are likely the proteins responsible for cross-reactive allergic reactions to peanuts, almonds, and hazelnuts (Stutius et al., 2010; Dreskin et al., 2021). Allergic reactions to oleosins from hazelnut and peanut oils have been confirmed as cross-reactive to sesame oil (Jappe and Schwager, 2017). Some plant proteins, notably those from tree nuts, can also cause pollen food allergy syndrome (PFS), an IgE-mediated allergy due to cross-reacting homologous proteins in pollens and various foods, including nuts, fruits, and vegetables (Weinberger and Sicherer, 2018). And allergy to chia seeds may cross-react with sesame allergy (Albunni et al., 2019).

Cross-reactivity also impacts animal-animal and animal-plant dietary proteins, which is highly relevant to the topic discussed here. The cross reactivity of food allergens from various animal and plant sources has been predicted for allergenic proteins that have mapped IgE-binding epitopes, T-cell epitopes, or both, with interesting findings. Some fish protein allergens share domains with chicken proteins, crustacean allergens share domains with those of mites and insects, and certain animal milk caseins share domains with soybean allergens (Kamath et al., 2023; Pereira et al., 2023). And the major shrimp allergen, tropomyosin, is a cross-sensitizing allergen in several edible insects due to its immunological similarities among crustaceans and arthropods, including insects (Liceaga, 2022).

Unless an animal protein is isolated from the expressing plant host and purified, mixtures of the animal protein and native plant proteins could exacerbate allergic reactions by exposing the consumer to higher doses of allergenic epitopes, or to allergens that may cause cross-reactive responses to foods to which the consumer is sensitized, possibly while being unaware of the risk. Such a risk will not escape regulatory scrutiny, and developers of products should address the allergenicity challenge proactively.

### 5.7 Approaches for mitigating dietary protein allergenicity

The most important way of mitigating allergenic risk is to prevent allergenic proteins expressed in various crops from inadvertently becoming mixed with non-GM varieties of the same crop. Failure to impose proper segregation and labeling could create a risk to consumers who are already reactive to the allergen from its original source.

Regulatory guidelines are in place in the US and elsewhere to minimize the risk of commingling GM and non-GM crops and their products. In more than 30 years of GM crop development, industry has voluntarily stopped research to express an allergenic protein in a commodity crop once they recognized the risk (Nordlee et al., 1996), and the US Department of Agriculture’s Animal and Plant Health Inspection Service (APHIS) has imposed fines for violations of permitting requirements for GM crops (7 CFR part 340) and taken action to segregate GM crops expressing non-dietary proteins that could have been commingled with non-GM crops (APHIS, 2024a). Notably, the governmental actions involved cases of human error or inadequate reporting vis-à-vis existing containment/confinement regulations (reviewed in Dietz and Muldoon-Jacobs, 2024) and were not related to the introduction of new dietary proteins in plants.

Although most dietary proteins are not allergenic to the majority of the population, what can be done to reduce allergenic risk to sensitive individuals? In addition to proper stewardship in compliance with regulatory guidelines (further discussed in Section 6), at least four main technical approaches are being explored for reducing the immunogenicity of dietary proteins. Some methods have been traditionally used by the food industry, while others are experimental and involve modification of the host and/or the dietary protein at the molecular level. Such approaches include: 1) physical or physicochemical methods to process native protein-containing food or its isolated native protein fraction; 2) genome engineering of the protein source (plant or animal) to produce hypo-allergenic proteins; 3) structure-based principles and methods to identify and modify allergenic epitopes in the target protein for subsequent expression of the de-immunized protein in a plant host; and 4) expression of human and humanized proteins as nutrient-balanced, non-allergenic options. Each individual approach has advantages and disadvantages, but it is possible to use a combination of techniques to achieve the desired goal.

The success of the “de-immunization” approach(es) can be verified by traditional methods, such as by immunological screening of the modified dietary protein against libraries of sera of individuals with clinically defined allergies to the original protein, enhanced by the availability of multiplex allergen microarrays (Tuppo et al., 2022), or by newer methods such as mass spectrometry, as has been practiced in the Food Protein Allergen Program at the U.S. National Institute of Standards and Technology (NIST).

Although a detailed discussion of the various methods that can be applied to mitigate allergenicity and/or de-immunize proteins is beyond the scope of this review, the major approaches that have already been applied to reduce dietary protein immunogenicity, or that could be applied to de-immunize yet-to-be defined dietary proteins, are summarized here and in **Table 4**. Conceptually, and if necessary, these de-immunization approaches could be applied to the specific animal proteins being developed in the efforts summarized in **Table 1** and future targets, and to the potential de-immunization of co-expressed animal/plant protein mixtures in plant tissues or seeds.

**Table 4:** Methods Available for De-Immunization of Dietary Proteins.

Method	Examples and Comments	Representative References
Processing	Disruption of the allergenic epitopes on proteins by physico-chemical methods. Examples include heating, acidic extraction, pressure, enzymatic digestion, hydrolysis, fermentation, exposure to ultrasound, cold plasma, ohmic heating.	Pereira et al., 2020, 2021; Day et al., 2022; Pereira et al., 2023; Wiederstein et al., 2023
Molecular Genetic	Genome-level engineering techniques to control the expression of allergenic dietary proteins in the source organism	
	Plants: Examples with plant proteins include transgene-induced gene silencing, RNAi silencing, and CRISPR/Cas9 (soy, wheat, peanut, rice proteins).	Mahler and Goodman, 2017; Sánchez-León et al., 2017; Sugano et al., 2020
	Plants: Glycoengineering of host plants to prevent plant-type glycan addition to proteins of interest (multiple hosts)	Montero-Morales and Steinkellner, 2018; Eidenberger et al., 2023
	Animals: Examples with animal proteins include zinc-finger nucleases and CRISPR/Cas9 (cow and ewe milk proteins).	Oishi et al., 2016; Zhou et al., 2017; Sun et al., 2018
Structure-Based	Molecular-level modification of allergenic epitopes in proteins. Many allergenic regions of dietary proteins have been mapped and results are available in several databases. Proteins devoid of or containing altered allergenic regions are then expressed in host systems to yield hypo-allergenic products	
	Protein Allergen Databases	AllergenOnline Allermatch AllFam Pfam (hosted by InterPro)
	Research and Feasibility Studies	van Ree et al., 2000; Scheurer and Sonnewald, 2009; King et al., 2014; Broekman et al., 2015; Hayes et al., 2015; Sanchez-Trincado et al., 2017; Callaway, 2020; Zinsli et al., 2021
Expression of Human Dietary Proteins	Expression of human milk proteins to obviate allergenicity concerns. Examples include recombinant human lactoferrin, transferrin, albumin, lysozyme.	Huang et al., 2002; Nandi et al., 2002, 2005; Zavaleta et al., 2007; Zhang et al., 2010

#### 5.7.1 Process-based protein de-immunization

Dietary protein processing is already widely practiced in the food industry, so ease of implementation at scale for de-immunizing animal proteins expressed in plants is a clear advantage of this option. However, the results are not always predictable and may be protein- or protein/food matrix-specific. Processing and cooking can exacerbate dietary protein allergenicity as well as reduce it (Day et al., 2022). Traditional food processing methods, including heating, acidic extraction, pressure, enzymatic digestion, hydrolysis and fermentation, as well as newer methods such as exposure to ultrasound or exposure to cold plasma, can alter the structure of allergenic proteins and alter both the nutritional value and allergenicity; the latter by disrupting the organization of IgE-binding epitopes (reviewed in Wiederstein et al., 2023). Some newer food processing technologies, such as ohmic heating (a process of heating food by passing an electric current directly into the food), can alter the ratio of monomeric to aggregated forms of β-lactoglobulin from milk whey (Pereira et al., 2020) as well as the properties of some soybean proteins, thereby reducing their allergenicity (Pereira et al., 2021).

#### 5.7.2 Genome-level engineering of the protein source organism and/or recipient host plant

Genome engineering has already been applied to modify plants and animals in attempts to lower the allergenic potential of their dietary proteins, and these efforts have produced encouraging results. For example, various molecular genetic techniques have been used in plants to control expression of genes encoding major allergenic proteins (Mahler and Goodman, 2017), including transgene-induced gene silencing (co-suppression) of Gly m Bd 30K in soybean; antisense gene silencing of 14- to 16-kDa allergens (α-amylase/trypsin inhibitor) in rice; RNAi silencing of Ara h 2.01, Ara h 2.02 and Ara h 6 in peanut; and various RNAi-based gene silencing attempts to reduce allergens in apple, tomato and carrot (Mahler and Goodman, 2017). CRISPR/Cas9 methods have also been successfully applied for genome engineering to reduce or even eliminate the expression of plant allergenic proteins, including reduction of soy allergens (Sugano et al., 2020) and generation of low-gluten lines of wheat (Sánchez-León et al., 2017). The same methods could be employed to modify the allergenicity of proteins in crops used as hosts for expression of animal proteins.

With animal dietary proteins, genetic regulation methods including zinc-finger nucleases (ZFNs) and CRISPR/Cas9 have been used in attempts to reduce allergenic milk proteins. β-lactoglobulin, the primary component of bovine and ovine milk whey proteins, is an important allergen because of its absence from human milk. In one study, a β-lactoglobulin gene knockout cow was generated using ZFN technology to yield β-lactoglobulin-free milk with significantly less IgE binding in cow’s milk-allergic individuals compared to unmodified whole milk (Sun et al., 2018). Similarly, in a separate study, CRISPR/Cas9 was used to generate goats with significantly lower levels of β-lactoglobulin in their milk (Zhou et al., 2017). For avian proteins, CRISPR/Cas9 has been used to target two hen egg white genes, ovalbumin and ovomucoid, to yield birds whose offspring expressed mutant protein (Oishi et al., 2016).

Although these studies suggest that crops and livestock with reduced content of allergenic proteins may be commercially developed, the expression or co-expression in plants of animal proteins that may be allergenic calls for a different solution, as it would be counter-intuitive to knock out the gene for the protein that one is attempting to express. Solutions may be found in post-harvest processing (already discussed), and in molecular modification of a protein’s allergenic epitopes and expression of the modified gene in a plant host to yield a hypo-allergenic product.

#### 5.7.3 Structure-based protein de-immunization

The goal of this approach would be to modify the allergenic epitopes of target proteins to make them non- or at least hypo-allergenic, so that the animal protein expressed in the plant host could offer improved nutritional properties as well as higher safety. There is historical precedent for this approach in the biopharmaceutical industry, where the de-immunization of biologic drugs was essential to their success (Baker et al., 2010). The approach is not without challenges, as changing the allergenic epitopes in proteins might negatively impact nutritional value, protein folding and functionality, protein expression level and other key properties that have yet to be understood.

Nevertheless, more than 20 computational tools have been used to identify T- and B-cell allergenic epitopes on proteins, with associated tools to predict the impact on the protein’s structure and functionality (reviewed in Hayes et al., 2015; Callaway, 2020; Zinsli et al., 2021). Many common structural, functional and biochemical properties in both plant and animal proteins have been identified and such efforts have enabled the assembly of protein allergen databases. For example, AllergenOnline, the Food Allergy Research and Resource Program (FARRP) database maintained by University of Nebraska – Lincoln, provides access to a peer-reviewed allergen list and sequence-searchable database intended for the identification of proteins that may present a potential risk of allergenic cross-reactivity. Similarly, the Allermatch database, maintained by Wageningen University, enables comparison of the amino acid sequence of a protein of interest with sequences of allergenic proteins, as well as prediction of the potential allergenicity of proteins by bioinformatics approaches, per the 2003 Codex Alimentarius/FAO/WHO recommendations. These and other databases continue to expand in content and could be instrumental in guiding the structure-based development of safer animal protein/plant protein dietary combinations.

#### 5.7.4 Humanization

Another approach to producing mammalian hypo-allergenic dietary proteins in plants would be to use human or humanized homologs. For example, human milk contains many bioactive components, with casein, α-lactalbumin, lactoferrin, secretory immunoglobulin IgA, lysozyme, and serum albumin comprising the major protein fractions. One could argue that there is no need to express in plants animal versions of milk proteins, some of which are allergenic, for nutritional or therapeutic uses when nutritionally more appropriate, fully functional, and non-allergenic human versions are available. Initial steps in this direction were successful more than 20 years ago, and included expression of human lactoferrin, transferrin and lysozyme in rice grains (Huang et al., 2002; Nandi et al., 2002, 2005; Zavaleta et al., 2007; Zhang et al., 2010). Such efforts could be revisited to express human milk and other proteins in various crops. However, with the exception of human milk proteins, consumption of other plant-made human proteins, such as the heme-protein myoglobin, might be restricted to therapeutic applications [i.e., oral dietary iron supplementation (Carlsson et al., 2020)].

## 6 Regulatory considerations

### 6.1 Current regulatory framework

As described, the novel PMF approach offers potential benefits in terms of sustainability, scalability, and ethical considerations compared to traditional agriculture. However, the regulatory oversight of plant expression of animal proteins is a complex issue that should be evaluated in a science-based, risk proportionate way. Under the Coordinated Framework for the Regulation of Biotechnology (Coordinated Framework), the U.S. Department of Agriculture (USDA), the U.S. Environmental Protection Agency (EPA) and the FDA have outlined roles and responsibilities on biotechnology product oversight, including genetically GM plants like those developed via PMF methods. USDA, through its Animal and Plant Health Inspection Service (APHIS), evaluates GM crops for their potential plant pest risk under the Plant Protection Act (PPA). APHIS ensures that GM plants are safe for import, movement, cultivation, and release into the environment, including those engineered to produce animal proteins. Product developers can request a regulatory status review (RSR) from APHIS. A plant developed using genetic engineering, determined by APHIS unlikely to pose a plant pest risk will not require regulation (7 CFR part 340). The first PMF product response was published in April 2024, determining that a ‘soybean developed using genetic engineering for accumulation of a meat protein’ was unlikely to pose a plant pest risk, and thus, is not subject to regulation under PPA (APHIS, 2024b). This decision includes progeny of the modified plant derived from crosses with other GM plants or other non-modified plants. In certain cases, the EPA may also play a role in regulating plant-produced animal proteins. EPA’s authority under the Federal Insecticide, Fungicide and Rodenticide Act (FIFRA) may be triggered if the proteins are engineered to produce substances considered pesticides, i.e., plant-incorporated protectants, or if they involve other genetic modifications that generate substances subject to regulation under FIFRA. The FDA regulates food, including food derived from GE sources, under the Federal Food, Drug, and Cosmetic Act (FD&C Act). Plant-produced animal proteins intended for human, or animal consumption fall under FDA’s oversight to ensure their safety, proper labeling, and compliance with food safety standards.

Under the Coordinated Framework, the FDA’s regulatory authority primarily focuses on ensuring the safety and proper labeling of food derived from GE plants. The agency assesses whether GE foods are equivalent to their conventional counterparts in terms of composition, nutritional value, and safety. FDA’s assessment aims to ensure that foods from GE plant-derived foods are as safe for human and animal food and feed consumption as their conventional counterparts and do not pose risk to public health. To carry out this mission, FDA operates a voluntary consultation process that offers developers an opportunity for premarket engagement with FDA on new products, by way of either a voluntary premarket consultation or voluntary premarket meetings (Food and Drug Administration, 2024). FDA’s voluntary premarket Plant Biotechnology Consultation Program, inclusive of New Protein Consultations, provides developers who ‘intend to commercialize food from a new plant variety’ an opportunity to discuss with the agency safety, nutritional and other relevant regulatory topics about the food. FDA provides feedback to the developer on the data that would be important for an assessment. FDA’s voluntary premarket meetings are available to a developer when a voluntary consultation is not necessary based on the characteristics of the food or the plant from which it is derived. FDA’s authority under the FDCA also extends to substances added to food and provides a number of mandatory opportunities for premarket consultation and/or approval for those.

### 6.2 Modernization and clarification of the regulatory framework for plant molecular farming

The current regulatory landscape governing PMF products requires clarity and modernization to ensure both innovation and safety. In this context, a recent letter from the FDA underscores a precautionary attitude towards an updated framework to facilitate the development and commercialization of PMF products (Food and Drug Administration, 2023). The FDA’s letter emphasizes the importance of establishing a clear and predictable regulatory pathway for developers acknowledging the advancements in biotechnology and increasing complexity products. However, in emphasizing the uncertainties and challenges in the regulatory process, this letter highlights the evolving technology space, with the possibility of increased uncertainty for developers regarding data requirements, safety assessments and labeling considerations. The lack of clarity and predictability in this system can impact developers by increasing time, resources and uncertainty in the review process when bringing products to market and frustrate the types of investments that enable transition from research and development to commercialization.

As laid out in the recent *Plan for Regulatory Reform under the Coordinated Framework for the Regulation of Biotechnology* (2024), FDA and USDA identified their intention to work together to examine stewardship mechanisms for PMF products. In this report, the agencies plainly signal that these stewardship mechanisms ‘may be led by developers or third parties with advice’ from the agencies. Clear communication and collaboration among regulatory agencies, industry stakeholders, and researchers are essential to facilitate the responsible development and commercialization of new technologies. Continued dialogue and engagement will be necessary to address emerging regulatory challenges, develop and facilitate stewardship mechanisms and promote innovation in this rapidly evolving field.

### 6.3 Guidelines for innovation

Clear regulatory guidelines are essential for providing industry stakeholders with certainty and predictability, enabling them to navigate complex regulatory landscapes effectively, as mentioned above. Science-based guidelines ensure that regulatory decisions are grounded in rigorous scientific evidence and risk assessment principles, minimizing the potential for bias or arbitrary decision-making, enhancing credibility and public trust in regulatory processes. Risk-proportionate guidelines allow regulatory agencies to prioritize resources and interventions based on the level of risk posed by different products or activities, optimizing regulatory efficiency and effectiveness while safeguarding public health and environmental integrity (Jones and Baumgartner, 2004; Schot and Steinmueller, 2018; Buchman et al., 2024).

Innovation must be balanced with robust safety standards to mitigate potential risks to human health and the environment. Innovation drives advancements in production efficiency, product quality and sustainability as mentioned in this article, enhancing the industry’s competitiveness and resilience in addition to the climate’s resilience. Adherence to safety standards not only ensures safety of products but fosters consumer confidence and market acceptance. Global and domestic stewardship practices promote sustainable development, environmental conservation, and equitable access to agricultural innovations, enhancing the industry’s credibility and social responsibility.

Food safety and allergen management are likewise important to address when fostering responsible development of PMF technologies and products. Comprehensive risk assessment methodologies exist and should be implemented in the evaluation of the potential hazards and benefits associated with PMF technologies. A recent critical assessment of PMF safety revealed that risk factors can be controlled by current practices (Buyel, 2023).

Federal agencies that are responsible for assessing scientific evidence to set public policy must also productively and truthfully convey to the public their remit and associated research. Public trust in information and knowledge provided by agencies is key to the ability of an agency to work and serve the public interest (Kowitt et al., 2017). For emerging technologies, like expression of animal proteins in plants, public trust in federal regulators is vital because the public relies on information from external sources, including government agencies (Gupta et al., 2020). Understanding public values and attitudes towards a particular technology may help policymakers develop guidance for emerging biotechnologies (Buchman et al., 2024). Broadly, taking precautionary steps to stifle an innovative product- or category of products- might impact public knowledge and awareness, risking the ability for an agency to take advancing and innovative approaches for future technologies not yet imagined.

## 7 Consumer acceptance of novel protein sources

It has been noted that consumers tend to more readily accept new medicines derived from GM organisms than GM-derived novel foods or ingredients; however, trends in consumer perception and acceptance of GM products may be evolving. A 2017 survey assessed public acceptance of GM foods and GM medicine, taking into consideration the level of consumer awareness and understanding of GM methodologies (Olynk Widmar et al., 2017). The results of that survey generally reinforced findings of past studies and found that people are more willing to accept the use of GM technology for human medicine and human health applications (62% and 68% respectively) than for livestock production, grain production, or fruit and vegetable production (44%, 49% and 48% respectively). Nevertheless, the fact that about one in two consumers surveyed were accepting of GM technology being applied to improve grains, fruits and vegetables is notable. A second finding from the Olynk Widmar et al. study was that acceptance of GM technologies was directly correlated with consumer awareness and understanding of the technologies.

A 2021 bibliometric analysis of 543 journal articles published from 1981 to 2021 identified that there is public divide in acceptance of GM foods in both developed and developing countries (R et al., 2022), consequently, there is a large gap between the increasing acceptance of GM crops for cultivation compared to what is available in the global market (Lucht, 2015), specifying that in order to reach developing countries in which food insecurity is rampant, general public acceptance and proper policies for these technologies are urgent (R et al., 2022).

Many studies in recent years have identified that public support for biotechnology in agriculture like GM crops or gene drive might be influenced by the decision of a government to do nothing, ban, or approve cultivation or deployment (R et al., 2022; Buchman et al., 2024). Additionally, when the potential benefits of a technology are clearly stated, public support increases (Caputo et al., 2020; Feint and Poortvliet, 2020; R et al., 2022; McFadden et al., 2024).

A public opinion poll conducted by Morning Consult on ‘Views of Agricultural Biotechnology’ found that some of the most important benefits of agricultural and industrial biotechnology tools are ‘increase the nutritional value of the foods you eat’ (83% important), ‘grow our economy through new technologies and industries’ (80% important), and ‘provide more plant-based alternatives to animal products, including meat and milk’ (66% important). This same polling identified that 66% of voters agree that the federal regulatory approval process should be modernized to allow agricultural biotechnology developers to commercialize innovative products expeditiously (Morning Consult, 2023).

In a recent survey conducted by the Edelman Trust Institute, on ‘Insights for the Food Sector’ identifies that implementation of technology is as important as invention, and that when there is mismanagement of these new technologies, there is a higher likelihood of societal resistance (Edelman Trust Institute, 2024). In the U.S., 24% of the population has high confidence that GMO foods will lead to a better future. Globally, those who are less than enthusiastic about the use of GMO foods identified that they would feel more positive if they understand it better (57%), see the benefits to society (54%), and see experts endorse it (51%) (Edelman Trust Institute, 2024).

These findings suggest that proper public education about the safety and benefits of new GM foods, including the ones discussed herein, may be key to their acceptance, and underscores the importance of science- and fact-based transparent communications from all stakeholders involved in food production, regulation, distribution and consumption. However, there is a gap in knowledge specific to PMF food products and its eventual place in the hands of consumers and there will likely be a need for additional survey and public perception research specific to questions and education surrounding PMF products.

## 8 Observations, conclusions and outlook

Current practices for producing animal meat are likely unsustainable in the long term and exacerbate climate change including global warming (FAO, 2017). A key reason for such negative environmental and resource impacts is that the efficiency of converting plant matter and water into animal protein is low. Poultry production requires 2 kg of feed per kg of protein produced (2:1 feed conversion ratio), with consumption of >4,000 L water/kg protein produced. It is even lower for mammalian meat, with a conversion ratio of 3:1 (kg feed/kg protein) and 6,000 L of water for pork, and is even worse for beef, which requires 8 kg feed per kg protein, using 14,500 L of water in the process (Raftowicz, 2022). In addition, production of a single kg of beef protein requires 200 m^2^ of land area, and produces 2.8 kg of CO_2_, 114 g of methane, and 170 mg of ammonia emissions (Liceaga, 2022). Although efforts are underway to improve the efficiency and lessen the environmental impact of animal agriculture, plant-based production of animal proteins offers a more efficient, scalable, sustainable and ethical alternative.

As reviewed here, multiple methods to express animal and human proteins in plants have been implemented over the last three decades to produce therapeutic proteins and vaccines as well as food and feed ingredients and additives, cosmetics, and enzymes for food processing and biofuel production. These methods are directly applicable to the large-scale production of dietary proteins, including the specific animal proteins discussed here. Importantly, the materials and methods used in PMF have been surveyed and assessed to be generally safe and environmentally benign. Although PMF is an innovative approach for producing animal proteins, consumer acceptance of these new products and ingredients as well as the ease of approval of such products by regulatory agencies is necessary for marketplace entrance.

Risk from the expression of animal proteins in plant is low. Over 90% of all known food allergies come from only 9 types of food, and the careful selection of target proteins to express in a new host should minimize allergenic risk from dietary exposure. If the animal protein is already consumed in its native form, its allergenic potential will likely be known, although the potential for new or cross-reactive allergens formed by post-translational modifications of the animal protein in the plant and/or the interaction of the animal protein with other proteins or components in the host plant matrix will need to be evaluated.

In summary, biotechnology is a collection of important tools for achieving global food security, offering innovative solutions to address key challenges in agricultural production, resource management, food quality and safety. PMF and related methods enable the precise modification of crops to produce protein-rich ingredients that can be utilized in alternative protein products, further diversifying protein sources and enhancing sustainability. Continuing research on production, resource management, quality, and stewardship as well as producer and consumer sentiment will be important for the ultimate PMF product market. The materials and methods used in PMF have been developed and optimized over several decades and are considered safe if properly applied and consistent with existing regulatory statutes, and so are the products they produce.
